# Os autores respondem

**DOI:** 10.1590/0102-311XPT104324

**Published:** 2024-09-09

**Authors:** Ester Paiva Souto, Celita Almeida Rosário, Michelle Vieira Fernandez, Priscila Cardia Petra, Gustavo Correa Matta

**Affiliations:** 1 Centro de Estudos Estratégicos da Fiocruz Antonio Ivo de Carvalho, Fundação Oswaldo Cruz, Rio de Janeiro, Brasil.; 2 Escola Nacional de Saúde Pública Sergio Arouca, Fundação Oswaldo Cruz, Rio de Janeiro, Brasil.; 3 Universidade de Brasília, Brasília, Brasil.; 4 Instituto Aggeu Magalhães, Fundação Oswaldo Cruz, Rio de Janeiro, Brasil.

Gostaríamos de agradecer pelo interesse demonstrado em nosso estudo *Hesitação Vacinal Infantil e COVID-19: Uma Análise a partir da Percepção dos Profissionais de Saúde* ^1^ e pela oportunidade de dialogar sobre os pontos levantados em sua carta ^2^. Apreciamos profundamente as observações detalhadas e as críticas construtivas apresentadas, que destacam aspectos importantes do nosso trabalho. Essas contribuições são valiosas para a excelência científica e enriquecem significativamente o debate sobre a hesitação vacinal. Os comentários a seguir foram elaborados com o intuito de esclarecer alguns pontos e ampliar o entendimento coletivo sobre esse fenômeno complexo e multidimensional.

## Conflitos de interesse e crise de confiança na ciência

Concordamos que a desconfiança como um fator de hesitação vacinal precisa ser contextualizada para ser abordada de forma adequada. A desconfiança nas vacinas, nas instituições de saúde e na ciência não é homogênea, ela varia significativamente de acordo com contextos históricos, culturais, sociais e individuais. Por exemplo, a desconfiança pode ser influenciada por narrativas contemporâneas de desinformação disseminadas por intermédio de redes sociais e outros meios.

Reconhecemos que o medo associado à vacinação é multifatorial e com fundamentações variadas que vão além das apresentadas em nosso estudo. Porém, nossas produções não detêm um caráter exaustivo acerca da compreensão de como as opiniões dos profissionais são formadas, sendo certo que não descartamos os conflitos de interesses na ciência e suas consequências para políticas de vacinação.

Infelizmente, devido às limitações editoriais, entre outros motivos expostos a seguir, não foi possível expandir essa análise no artigo. No entanto, entendemos a importância dessa abordagem e consideramos que futuras pesquisas devem explorar essas dimensões com maior profundidade para uma compreensão mais abrangente do fenômeno.

## Agrupamento dos profissionais de saúde na análise dos dados

Entendemos que médicos, enfermeiros, técnicos de enfermagem e agentes comunitários de saúde têm interações distintas com a comunidade, o que pode influenciar suas percepções e práticas em relação à vacinação. No entanto, para os fins deste estudo, optamos por tratar esses grupos de forma unificada, especialmente considerando o contexto da atenção primária à saúde (APS), em que as funções e percepções na temática estavam alinhadas em nossos resultados. Além disso, especificamente para o objetivo de pesquisa deste artigo, o agrupamento se mostrou mais interessante para a análise de conteúdo pelo número de profissionais entrevistados. Mesmo assim, demos importância à indicação de quais profissionais pertencem as falas expostas, no intuito de que o leitor possa ter os seus próprios *insights*. Uma análise segmentada poderia fornecer *insights* diferentes.

## Sobre o método e seleção de temas para a discussão

Nossa pesquisa realizou uma análise temática com base no conteúdo das entrevistas conduzidas com profissionais de saúde. Observamos e interpretamos as narrativas coletadas, atribuindo códigos indutivos definidos pela equipe de pesquisadores a partir da análise do material empírico. Os temas de conflitos de interesse e crise de confiança na ciência e debates sobre a atuação dos conselhos profissionais não foram abordados nos códigos escolhidos para integrar o artigo. Dessa forma, a equipe de pesquisadores priorizou a percepção dos profissionais da saúde no intuito de visibilizar suas experiências ao longo do período. Além disso, nosso grupo de pesquisa está atualmente desenvolvendo um artigo específico sobre a influência dos conselhos profissionais na hesitação vacinal, o que reforça nossa compreensão da relevância desse tópico. Acreditamos que a análise futura desse tema contribuirá significativamente para o entendimento das dinâmicas de hesitação vacinal e para a formulação de políticas mais eficazes.

## Ampliar as recomendações do estudo

Concordamos com a importância de ampliar as recomendações do estudo. Nosso grupo de pesquisa também reconheceu essa demanda e está desenvolvendo uma publicação que aborda estratégias para dialogar com as narrativas hesitantes, compreendendo sua produção, sentidos e contradições. Além disso, estamos criando um curso autoinstrucional sobre hesitação vacinal voltado para profissionais da APS, com o objetivo de equipá-los com ferramentas e conhecimentos para enfrentar esse desafio. Acreditamos que essas iniciativas contribuirão para a implementação de estratégias mais eficazes e adaptadas às realidades locais, promovendo uma maior adesão às vacinas.

## Considerações finais

A hesitação vacinal é uma questão complexa e multidimensional que exige uma abordagem interseccional para ser plenamente compreendida. Esperamos que o debate contínuo e colaborativo leve à formulação de políticas que aumentem a adesão à vacinação, promovendo uma saúde pública mais robusta e resiliente. Agradecemos mais uma vez pela oportunidade de discutir nosso trabalho e confiamos que essas considerações contribuirão para enriquecer o debate científico sobre a hesitação vacinal, fomentando avanços significativos na área.
